# Depression, anxiety, and stress in partners of Australian combat veterans and military personnel: a comparison with Australian population norms

**DOI:** 10.7717/peerj.2373

**Published:** 2016-08-25

**Authors:** Gail V. MacDonell, Navjot Bhullar, Einar B. Thorsteinsson

**Affiliations:** Psychology-School of Behavioural, Cognitive and Social Sciences, University of New England, Armidale, New South Wales, Australia

**Keywords:** Dyadic adjustment, Combat veterans, Caregiving distress, Mental health, Partners of veterans, PTSD

## Abstract

Partners of Australian combat veterans are at an increased risk of experiencing mental health problems. The present study provides a comparative analysis of the mental health of partners of veterans with that of the Australian normative data. To compare different types of groups of partners, the study samples comprised: (a) partners of Australian combat veterans (Sample 1: *n* = 282, age *M* = 60.79, *SD* = 5.05), (b) a sub-sample of partners of Australian combat veterans from the previous sample (Sample 2: *n* = 50; *M* = 60.06, *SD* = 4.80), (c) partners of Special Air Services Regiment (SASR) personnel (Sample 3: *n* = 40, age *M* = 34.39*SD* = 7.01), and (d) partners of current serving military (non-SASR) personnel (Sample 4: *n* = 38, age *M* = 32.37, *SD* = 6.20). Respondents completed measures assessing their reported levels of depression, anxiety, and stress. Samples 1 and 2 comprised partners of Australian military veterans who reported significantly greater symptoms of depression, anxiety, and stress than the comparative population norms. The sample of SASR personnel partners (Sample 3) reported significantly lower levels of depression and anxiety, whereas the sample with non-SASR personnel partners (Sample 4) reported a significantly greater stress symptomatology than the comparative norms. Number of deployments was found to be associated with depression, anxiety, and stress in partners of non-SASR veterans (Sample 4). Lessons and protective factors can be learnt from groups within the current military as to what may assist partners and families to maintain a better level of psychosocial health.

## Introduction

Partners of military and combat veterans^1^ 1In this paper, we referred to all Military personnel as combat veterans.are at a risk of experiencing higher levels of mental health distress symptomatology (Alessi et al., 2001). Progress has been made in research of partners of veterans regarding their psychosocial outcomes and the major issues they face (e.g., Beckham, Lytle & Feldman, 1996; Calhoun, Beckham & Bosworth, 2002; MacDonell et al., 2014; Outram et al., 2009; Renshaw & Campbell, 2011; Renshaw & Caska, 2012; Solomon et al., 1991; Westerink & Giarratano, 1999). Evidence suggests that partners of veterans with Posttraumatic Stress Disorder (PTSD) showed higher levels of emotional distress than those in the non-military population (Dekel et al., 2005a), and that partners of veterans with PTSD experienced higher levels of caregiver burden and lower levels of psychological adjustment than those partners whose veteran did not have PTSD (Calhoun, Beckham & Bosworth, 2002). The majority of this research has compared the partners of veterans who reported to have PTSD with that of partners without PTSD. However, no research to date has compared the partners of different services (e.g., regiments) personnel; therefore, the present paper examined the mental health of partners of veterans from three different military services and compared them with the Australian population norms.

There is a growing interest in understanding the relationship between veterans’ deployment stressors and exposure to combat and their partners’ risk for mental health and dyadic adjustment problems (Calhoun, Beckham & Bosworth, 2002). The personal and family relationships of veterans are often marked by considerable distress and dyadic maladjustment (MacDonell et al., 2010; MacDonell et al., 2014; Outram et al., 2009). Findings from the United States, Israel, Holland, Croatia, Iran (Calhoun, Beckham & Bosworth, 2002; Dekel et al., 2005a; Dirkzwager et al., 2005; Frančeikovišć et al., 2007; Salimi et al., 2006) and Australia (MacDonell et al., 2010; MacDonell et al., 2014; Outram et al., 2009; Westerink & Giarratano, 1999) have suggested that partners of combat veterans have a significantly higher risk of developing psychosocial problems as a result of living with and caring for their veterans, particularly those veterans with PTSD. Moreover, it has been shown that the psychosocial functioning of partners is poorer than the general population overall (Beckham, Lytle & Feldman, 1996; MacDonell et al., 2010; Westerink & Giarratano, 1999). Research has found that the greater the symptoms of PTSD of the veterans, the greater the distress in their partners (Dekel, Solomon & Bleich, 2005b; Pearlin et al., 1990). The long-term effects of PTSD have shown to be labile, but indicate that the detrimental effects are complex, deep and enduring (Solomon et al., 2014; Solomon & Mikulincer, 2006).

Research has also shown that multiple deployments for veterans tend to lead to higher rates and severity of PTSD (Hoops, 2012; Kline et al., 2010) and that the military lifestyle can have negative outcomes on the family, particularly the spouse or partner (Burrell et al., 2006). Separation (not only from deployment), unpredictable duty hours, frequent relocations and single parenting (parenting while the veteran is away either being deployed or on training courses) are just a few of the stressors that face partners of veterans on a regular basis (Padden, Connors & Agazio, 2011). Moreover, attempting to build a career while being a partner of a veteran is difficult, with some suggesting that existing gender inequality in the workplace gives these partners a dual disadvantage (Doherty, Patton & Shield, 2015). There is also evidence that partners’ behavioral health is becoming more problematic (Ahmadi & Green, 2011), including increases in drug and alcohol use. Research has started to recognise that the partner of a veteran plays a crucial role in the health of the veteran and the couple’s relationship (Lewis, Lamson & Leseuer, 2012). However, there is limited research into the long-term effects on the partners and families, over the lifespan of the relationships (Link & Palinkas, 2013), therefore, more research is needed to examine the possible negative feedback loop between family factors that affect the combat veterans and their families.

The present paper investigated the degree of psychological distress by examining levels of depression, anxiety and stress in partners of veterans serving in three different military services, and compares them with norms for the Australian population. We specifically examined the similarities and differences between different samples of partners of veterans: (a) partners of veterans (POV) who have left the military (Samples 1 and 2); (b) current serving Special Air Services Regiment (SASR, Sample 3); and (c) currently serving military who are partners of non-SASR veterans (Sample 4). A description of all the four samples is provided in Method section. Previous research also suggests that a greater number of deployments is associated with higher levels of PTSD symptomology in combat veterans which, in turn, may have a negative impact on their partners’ mental health outcomes. Although, no official data on deployments is currently available, the anecdotal evidence suggests that SASR personnel experience more deployments than other military personnel. Therefore, we predicted that the partners of SASR veterans would report significantly greater psychological distress as compared with other samples of partners.

## Method

### Participants

#### Sample 1

Participants were 282 female partners of Australian veterans (i.e., partners of veterans (POV)). These participants were members of the Partners of Veterans Association of Australia (PVA). Membership in this association is open to all partners of current and former partners (those who may have separated from their veteran spouse or who are widowed) who served in the theatre of war or campaign, or who are currently serving in any peacekeeping, peace-making, or operational service. Meetings in different areas are usually held on a monthly or more regular basis as a way of support and air any problems the partners may have. Participants were recruited via the PVA newsletter and they completed a self report questionnaire. Partners’ ages ranged from 43 to 83 years (*M* = 60.79, *SD* = 5.05). Their length of marriage ranged from seven to 60 years (*M* = 35.43, *SD* = 9.74). Participants reported that their veterans had served in the Air Force (12%), Army (82%), and Navy (6%). Just over half (53%) of the veterans had been long-term service personnel, with 44% being National Conscripts, 3% where National Conscripts who stayed on to become long term service personnel. The percentage of veterans who attended different conflict zones was 1% in World War II, 2% in Korea, 9% in Malaya/Borneo, 82% in Vietnam, 0.5% each in Somalia, Sinai, Kashmir, Gulf War 1 and East Timor, whereas 1% indicated ‘other’ as their first conflict. Of those who indicated that their veteran had been deployed in an active service zone more than once, 68% had been deployed twice, 9% three times, 9% four times and 4% five times.

#### Sample 2 (as second POV sample)

The first 50 participants who were still living with a veteran at the time of data collection were selected from Sample 1. POV female partners’ ages ranged from 43 to 70 years (*M* = 60.06, *SD* = 4.80). Their length of marriage ranged from seven to 47 years (*M* = 34.22, *SD* = 10.41). Of those five indicated that their veteran had been deployed in an active service zone more than once, 20 twice, five 3-times, five 4-times, and five 5-times. Ten did not indicate their partners’ number of deployments.

#### Sample 3

These were 40 female partners of Special Air Service Regiment (SASR) personnel. These partners were a convenience sample who completed an online survey over a period of two weeks following meetings discussing their issues. The SASR personnel partners’ ages ranged from 23 to 49 years (*M* = 34.39, *SD* = 7.01). The length of their relationship ranged from 1 to 30 years (*M* = 11.66, *SD* = 7.53). In Sample 3, two veterans had not been deployed, two had been deployed once, one twice, six 3-times, four 4-times, six 5-times, and two 7-times, five 8-times, four 9-times, two 10-times and six had been on deployed over 12-times.

#### Sample 4

This group consisted of 38 female partners of current Defence personnel (non-SASR). This was a convenience sample completing an online survey over a period of two weeks following meetings discussing their issues. Defence partners’ (non-SASR) ages ranged from 22 to 48 years (*M* = 32.37, *SD* = 6.20). The length of the relationship ranged from two to 23 years (*M* = 9.55, *SD* = 5.07). Ten defence personnel had been deployed once, seven twice, thirteen 3-times, one 5-times, one 7-times, one 8-times, and five had been deployed more than 12-times.

### Measures

#### The Depression Anxiety Stress Scales (DASS)

The 21-item DASS (Lovibond & Lovibond, 1995a; Lovibond & Lovibond, 1995b) was used to assess levels of depression, anxiety, and stress. This self-report scale comprises three subscales: depression, anxiety and stress. Each subscale consists of seven items, answered on a Likert like scale from 0 (“*Did not apply to me at all*”) to 3 (“*Applied to me very much, or most of the time*”). A sample item for Depression is “*I couldn’t seem to experience any positive feeling at all”*; for Anxiety: “*I was aware of dryness of my mouth*”, and for Stress: “*I found it difficult to relax”*. Low scores on these subscales reflect better mental health. Antony et al. (1998) validated the scale showing that depressive patients scored highest on the depression and stress subscales, whereas panic disorder patients scored highest on the anxiety subscale. The depression subscale measures dysphoria, hopelessness, anhedonia and inertia, whereas the anxiety subscale assesses autonomic arousal, skeletal musculature effects, situational anxiety and subjective experience of anxious effect (feelings of panic and fear). The stress subscale assesses difficulty in relaxing, impatience and chronic non-specific arousal (Lovibond & Lovibond, 1995a). The totals of each subscale were multiplied by two to reflect the scores of the DASS 42 and referenced to the normative data (Lovibond & Lovibond, 1995a).

For depression, anxiety, and stress, Cronbach’s *α*s for Sample 1 (POV) were .94, .90 and .91, respectively; for Sample 2 (second POV sample), *α*s were .86, .87 and .81, respectively; for Sample 3 (SASR), *α*s were .78, .62, and.62, respectively, and Sample 4 (non-SASR), *α*s were .96, .88, and .84, respectively.

### Procedure

Participants in Sample 1 (POV) were asked to complete a self-report questionnaire that was sent out in a national newsletter to partners of veterans, as part of a larger study (MacDonell et al., 2014). Both SASR and non-SASR (Samples 3 and 4) participants were convenience samples and were asked to fill out a questionnaire online over a 2-week period. Sample 2 participants who were still living with a veteran at the time of data collection were selected from Sample 1 to create a POV sample comparative in number with Samples 3 and 4. Ethics approval was given by Human Research Ethics Committee, University of New England, and approval number HE09/151. Participants gave their consent by either completing the hard-copy of the questionnaire or clicking a ‘proceed’ button on the online questionnaire after reading the information sheet for the study.

### Statistical analysis

All statistical analyses were run using SPSS version 22 using two datasets, one with Sample 1 (MacDonell, Bhullar & Thorsteinsson, 2016a) and one with Samples 2, 3, 4 combined (MacDonell, Bhullar & Thorsteinsson, 2016b). For the comparative analyses, a series of one-sample *t*-tests were conducted with Bonferroni adjustments where applicable. All missing data on study variables (<3%) were considered missing completely at random and were replaced with values computed by the expectation maximization algorithm in SPSS (Tabachnick & Fidell, 2001).

## Results

The reported levels of depression, anxiety, and stress were moderately higher, than Australian female normed values, in Sample 1 (Table 1) and Sample 2 (Table 2). Our results indicated that there were no statistical differences for depression, anxiety, and stress between Samples 1 and 2 (both POV samples).

**Table 1:** Summary statistics for DASS for Sample 1 partners of Australian combat veterans (POV; *n* = 282) and DASS normative data.

Measure	POV *M* (*SD*)	Norms *M* (*SD*)	*t*(281)	Mean difference	95% CI (Mean difference)	Hedges’ *g*	95% CI (Hedges’ *g*)
Depression	16.09 (12.58)	6.14 (6.92)	13.27^*^	9.95	8.47, 11.42	0.79	0.62, 0.96
Anxiety	12.61 (11.83)	4.80 (4.70)	11.08^*^	7.81	6.42, 9.19	0.66	0.49, 0.83
Stress	19.69 (11.71)	10.29 (8.16)	13.47^*^	9.39	8.02, 10.77	0.80	0.63, 0.97

**Notes.**

Norms based on Females’ normative data sample (*N* = 1,870), Lovibond & Lovibond (1995b). Hedges’ effect size: small (0.2) medium (0.5) and large (0.8). Tests on the four groups were conducted using the Bonferroni adjustment alpha levels of .016 (.05/3).

* *p* < .001, two-tailed.

**Table 2:** Summary statistics for DASS for Sample 2 partners of Australian combat veterans (POV; *n* = 50) and DASS normative data.

Measure	POV *M*(*SD*)	Norms *M*(*SD*)	*t*(49)	Mean difference	95% CI (Mean difference)	Hedges’ *g*	95% CI (Hedges’ *g*)
Depression	18.84 (12.27)	6.14 (6.92)	7.32^*^	12.70	9.21, 16.19	0.44	0.27, 0.60
Anxiety	12.88 (11.00)	4.80 (4.70)	5.19^*^	8.08	4.95, 11.22	0.31	0.41, 0.48
Stress	22.68 (11.58)	10.29 (8.16)	7.56^*^	12.39	9.09, 15.68	0.45	0.28, 0.62

**Notes.**

Norms based on Females normative data sample (*N* = 1,870) Lovibond & Lovibond (1995b). Hedges’ effect size: small (0.2) medium (0.5) and large (0.8). Tests on the four groups were conducted using the Bonferroni adjustment alpha levels of .016 (.05/3).

* *p* < .001, two-tailed.

The findings for partners of Australian SASR members (Sample 3) showed that they had lower levels of depression and anxiety than the normative data (see Table 3). Comparing normative data of depression, anxiety, and stress levels in partners of Australian current serving non-SASR members (Sample 4) showed a small significant difference for stress. The data indicated higher levels of stress in the non-SASR members than in the normative data (see Table 4).

**Table 3:** Summary statistics for DASS for sample 3 Special Air Service Regiment (SASR; *n* = 40) and DASS normative data.

Measure	SASR *M* (*SD*)	Norms *M* (*SD*)	*t*(39)	Mean difference	95% CI (Mean difference)	Hedges’ *g*	95% CI (Hedges’ *g*)
Depression	4.15 (4.58)	6.14 (6.92)	−2.75^*^	−1.99	−3.46, −0.52	−0.16	−0.33, 0.00
Anxiety	2.45 (5.39)	4.80 (4.70)	−2.76^*^	−2.35	−4.07, −0.63	−0.16	−0.33, 0.00
Stress	9.65 (7.62)	10.29 (8.16)	−0.53	−0.64	−3.08, 1.79	−0.03	−0.20, 0.13

**Notes.**

Norms based on Females’ normative data sample (*N* = 1,870), Lovibond & Lovibond (1995b). Hedges’ effect size: small (0.2) medium (0.5) and large (0.8). Number of participants is 40 rather than 41 as one participant did not answer the DASS questionnaire. Tests on the four groups were conducted using the Bonferroni adjustment alpha levels of .016 (.05/3).

* *p* < .012, two-tailed.

**Table 4:** Summary statistics for DASS for Sample 4 of current serving non-Special Air Service Regiment (non-SASR; *n* = 38) and DASS normative data.

Measure	Non-SASR *M* (*SD*)	Norms *M*(*SD*)	*t*(37)	Mean difference	95% CI (Mean difference)	Hedges’ *g*	95% CI (Hedges’ *g*)
Depression	9.63 (10.04)	6.14 (6.92)	1.95	3.50	−0.14, 7.12	0.32	−0.14, 0.77
Anxiety	4.32 (5.63)	4.80 (4.70)	−0.53	−0.59	−2.33, −1.37	−0.09	−0.54, 0.36
Stress	14.58 (8.97)	10.29 (8.16)	2.94^*^	4.29	1.34, −7.24	0.48	0.02, 0.93

**Notes.**

Norms based on Females’ normative data sample (*N* = 1,870), Lovibond & Lovibond (1995b). Hedges’ effect size: small (0.2) medium (0.5) and large (0.8). Tests on the four groups were conducted using the Bonferroni adjustment alpha levels of .016 (.05/3).

* *p* < .012, two-tailed.

Sample 1 reported mean scores in the *moderate* range for depression (*M* = 16.09), anxiety (*M* = 12.61), and stress (*M* = 19.69), and the Sample 2 depression mean scores (*M* = 18.84), anxiety (*M* = 12.88) and stress (*M* = 22.68) were also in the moderate range. Sample 3 were in the non-clinical range for all categories: depression (*M* = 4.15), anxiety (*M* = 2.45), and stress (*M* = 9.65). Sample 4 reported values in the non-clinical range for depression (*M* = 9.64) and anxiety (*M* = 5.59), but marginally in the mild category for stress (*M* = 14.31). The severity ratings for DASS normative data (Lovibond & Lovibond, 1995a) are presented in Table 5.

**Table 5:** DASS 42 severity ratings taken from Lovibond & Lovibond (1995b).

Rating	Depression	Anxiety	Stress
Normal	0–9	0–7	0–14
Mild	10–13	8–9	15–18
Moderate	14–20	10–14	19–25
Severe	21–27	15–19	26–33
Extremely severe	28 +	20 +	34 +

As expected, Table 6 shows that Sample 3 reported a higher number of deployments than (a) Sample 2, Hedges’ *g* = 1.33 (95% CI [0.87–1.78]) and (b) Sample 4, Hedges’ (*g* = 0.77 [95% CI [0.31–1.22]). However, the correlations between the number of deployments and depression, anxiety, and stress measures were large and statistically significant for Sample 4 only, see Table 6.

**Table 6:** Mean and standard deviation for number of deployments for different samples and the correlation between deployments and depression, anxiety, and stress.

Sample	*n*	*M*	*SD*	*r*		
				Depression	Anxiety	Stress
Sample 2: POV	40	2.63	1.23	.16	.01	.13
Sample 3: SASR	40	6.40	3.89	.18	.04	.22
Sample 4: Non-SASR	38	3.95	4.03	.35^*^	.39^*^	.40^*^

**Notes.**

POV, Partners of veterans; SASR, Special Air Services Regiment

* *p* < .05, two-tailed.

## Discussion

The present study compared three of the major symptoms of psychosocial dysfunction (depression, anxiety and stress) in partners of Australian combat veterans with Australian normative data. It also examined some of the similarities and differences within different samples of those partners. To date, there has been no comparison of mental health symptomatology relative to normative data, nor have there been comparisons between different groups of partners of Australian veterans. The present paper provides a preliminary snapshot of the psychological distress experienced by these groups of partners of veterans. The results did not fully support our prediction that the more deployments a veteran had, the greater psychosocial dysfunction the partner would experience. Number of deployments was not related to depression, anxiety, or stress in the POV or the SASR samples. However, the higher number of deployments was associated with significantly greater levels of depression, anxiety, and stress in the partners of non-SASR veterans. This could indicate that factors related to number of deployments such as constant moving of location and poor social support satisfaction and networks may be the underlying factors affecting depression, anxiety, and stress in these partners.

Many researchers have found that the health of the partner impacts significantly on the health outcomes of the veterans (Ahmadi & Green, 2011) and that partners and family units are the principal support systems for veterans (Renshaw, Rodebaugh & Rodrigues, 2010). However, limited emphasis has been put on comparative analysis of the psychological health of partners relative to Australian normative data. Results indicated that those in the older mean age samples comprising partners of Australian veterans (Samples 1 and 2) reported greater symptomatology as compared with normative data than those in the younger samples comprising partners of currently serving military personnel (Samples 3 and 4). This may be from long-term caring responsibilities and the longer length of time of living with a veteran (Outram et al., 2009), and the results may have implications for support services for the partners and families of combat veterans. For example, preventative programs may be more appropriate for the younger partners, whereas more clinical and respite support services may be required for the older partners.

It should also be noted that partners of SASR members had lower levels of depression and stress, than the Australian normative data, although the effect sizes were small. Partners of SASR members have the ability to stay in the same location compared to the non-SASR partners. Some non-SASR partners can be relocated every two to three years from one side of Australia to another and have to form new relationships and support systems with each move. Constant relocation combined with multiple deployments may lead to higher levels of stress (Australian National Audit-Office, 2012). The majority of the SASR-cohort partners were professionals with a solid career. Constant relocations have shown to have a detrimental effect on partners of non-SASR career and employment opportunities (Australian National Audit-Office, 2012). This could serve as another life stressor for the partners. Our results show that current–serving non-SASR partners in this cohort reported elevated stress levels compared to the Australian normative data. This could reflect a constant state of hyperarousal in the partners, which reflects the issues and problems they experience in meeting their daily life demands (Lovibond & Lovibond, 1995b.).

### Limitations and future research

First, only a single measure (DASS) was used to examine mental health symptoms. Future research could include other measures to assess mental health problems as well as positive concepts such as psychological and physical well-being. Second, the present study used a convenience sample, which could potentially have biased the results. Third, the sample sizes were relatively small in Samples 2, 3, and 4 and Sample 3 had lower Cronbach’s alpha levels for the DASS subscales. Future research could examine several different concepts more carefully such as age, number of relocations, and career. Future research might also like to employ a large cohort of partners and veterans in a longitudinal study, thus examining outcomes such as suicide and prevention not only for veterans (e.g., Yi & Hong, 2015) but for their partners as well. More research needs to be done on partners of the different armed forces such as Army, Navy, Air Force and Australian Federal Police who serve overseas.

### Conclusion

Mental health of partners of Australian veterans is generally poorer in terms of greater depression, anxiety and stress levels than the comparative norms. However, partners of SASR personnel seemed to have lower levels of depression and anxiety than the comparative norms. Elevated levels of depression, anxiety, and stress appear to be associated with higher number of deployments in non-SASR partners but this relationship may be explained by other factors as this relationship did not hold in the POV or SASR samples.

##  Supplemental Information

10.7717/peerj.2373/supp-1 Supplemental Information 1 Screen shot of the information/consent form as shown before the online survey

## Additional Information and Declarations

### Data Availability

The following information was supplied regarding data availability: MacDonell GV, Bhullar N & Thorsteinsson EB (2016a). De-identified DASS norms POV282 [DATA]. figshare. Available at: http://dx.doi.org/10.6084/m9.figshare.3120697. MacDonell GV, Bhullar N, & Thorsteinsson EB (2016b). De-identified DASS norms three groups (POV50, SASR41, non-SASR38) [DATA]. figshare. Available at: http://dx.doi.org/10.6084/m9.figshare.3120676.
