# Supplementary figures and images for: Depression, anxiety, and stress in partners of Australian combat veterans and military personnel: a comparison with Australian population norms
Source: PeerJ. 2016 Aug 25;4:e2373. doi: 10.7717/peerj.2373 (PMC5012292; doi:10.7717/peerj.2373)

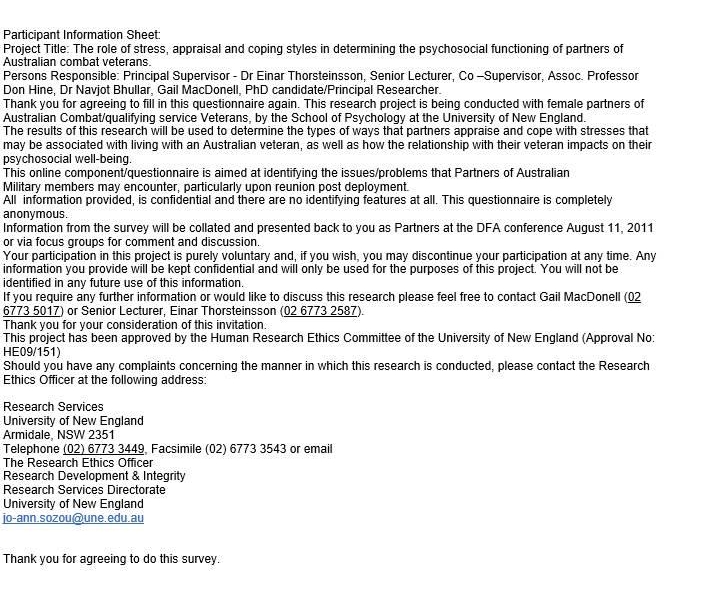

Supplement: Supplemental Information 1 [file peerj-04-2373-s001.jpg]
